# Initial risk perception and feeling of preparedness of primary care physicians regarding the COVID-19 pandemic in Belgium, France and Spain in February 2020

**DOI:** 10.1186/s12875-021-01588-5

**Published:** 2022-01-23

**Authors:** C Guerrisi, B Thomas, A Ordax Diez, D Van Cauteren, J E Lozano Alonso, S Moreels, A Falchi, T Vega Alonso, I Bonmarin, J Raude, A M Vilcu, T Hanslik, M Debin, L Rossignol, V Colizza, C Souty, T Blanchon

**Affiliations:** 1 Sorbonne Université, INSERM, Institut Pierre Louis d’Épidémiologie et de Santé Publique, IPLESP, F75012 Paris, France; 2 Dirección General de Salud Pública, Consejería de Sanidad, Castile and León, Valladolid, Spain; 3 Scientific Directorate of Epidemiology and public health, Sciensano, Brussels, Belgium; 4 EA 7310, Laboratoire de Virologie, Université de Corse, 20250 Corte, France; 5 Santé publique France, Saint-Maurice, France; 6 EHESP Rennes, Université Sorbonne Paris Cité, Paris, France; 7 Service de Médecine Interne, Hôpital Ambroise Paré, Assistance Publique - Hôpitaux de Paris, APHP, FR-92100 Boulogne Billancourt, France; 8 Université de Versailles Saint-Quentin-en-Yvelines, UVSQ, UFR de Médecine, FR-78000 Versailles, France; 9 Université de Paris, Département de Médecine Générale, Paris, France

**Keywords:** Primary Health Care, COVID-19, Pandemic, Anxiety, Health information, Management, Sentinel Surveillance

## Abstract

**Background:**

The knowledge of risk perceptions in primary care could help health authorities to manage epidemics.

**Methods:**

A European multi-center cross-sectional study was conducted in France, Belgium and Spain to describe the perceptions, the level of anxiety and the feeling of preparedness of primary healthcare physicians towards the COVID-19 infection at the beginning of the pandemic. The factors associated with the feeling of preparedness were studied using multivariate logistic regressions.

**Results:**

A total of 511 physicians participated to the study (response rate: 35.2%). Among them, only 16.3% (n=82) were highly anxious about the pandemic, 50.6% (n=254) had the feeling to have a high level of information, 80.5% (n=409) found the measures taken by the health authorities suitable to limit the spread of COVID-19, and 45.2% (n=229) felt prepared to face the epidemic. Factors associated with feeling prepared were: being a Spanish practitioner (adjusted OR=4.34; 95%CI [2.47; 7.80]), being a man (aOR=2.57, 95%CI [1.69; 3.96]), finding the measures taken by authorities appropriate (aOR=1.72, 95%CI [1.01; 3.00]) and being highly informed (aOR=4.82, 95%CI [2.62; 9.19]).

**Conclusions:**

Regarding the dramatic evolution of the pandemic in Europe in the weeks following the study, it appears that information available at this time and transmitted to the physicians could have given a wrong assessment of the spread and the severity of the disease. It seems essential to better integrate the primary care physicians into the information, training and protection channels. A comparison between countries could help to select the most effective measures in terms of information and communication.

**Supplementary Information:**

The online version contains supplementary material available at 10.1186/s12875-021-01588-5.

## Background

At the end of 2019, a new coronavirus (SARS-CoV-2), causing respiratory infections (COVID-19), emerged in China and further spread worldwide [1]. The number of reported cases increased steadily in Europe at the beginning of March 2020 [2], generating fears and anxiety among the general population and health care workers [3–5].

Previous health crisis due to coronaviruses (SARS in 2002/2003, MERS-CoV in 2012), or other infectious diseases (H1N1 pandemic influenza in 2009, Ebola in 2014) have shown the importance of collecting feedback of healthcare professionals [6–8]. Indeed, knowledge of primary healthcare professionals’ perceptions and behavior represents an essential tool for health authorities to implement control measures and communication campaigns [9]. Knowing perceptions and behavior at the beginning of the pandemic is particularly relevant as it enables health authorities to adapt and specifically target their actions.

Here we describe the perceptions, anxiety and feeling of preparedness of primary healthcare physicians towards the COVID-19 infection at the beginning of the pandemic in Europe, in three European countries: Belgium, France, and Spain.

## Methods

### Design and study population

A cross-sectional study was carried out in February-March 2020 among all the primary care physicians involved in European sentinel surveillance networks: “Sciensano” (Belgium), “Réseau Sentinelles” (France), and “Red Centinela Sanitaria” (Castile and León, Spain). The participants included were general practitioners (GPs) and pediatricians (only in Spain). Physicians were invited to participate in the study on a voluntary basis. The representativeness of Belgian, French and Spanish sentinel physicians has been previously studied [10–12].

### Data collection and study period

The questionnaire was built according to the literature [13, 14], and was validated by a panel of experts: members of the French national public health agency (i.e. Santé publique France), epidemiologists and biostatisticians, a general practitioner and a sociologist. The survey included 24 questions and addressed topics on (i) anxiety due to the ongoing pandemic (from the physician and the patient perspectives), (ii) practices’ changes (probability of seeing infected patients, repercussions on the consultations’ organization, anticipated preparation measures), (iii) information received from health authorities (regarding the COVID-19 epidemic in China, the risk for the local population, the case definition, the management of suspected cases), and (iv) feeling of preparedness. The detailed questionnaire is available in a supplementary file (see Additional file 1). To characterize healthcare professionals, demographic characteristics were collected: age, gender and the practice area. Physician practice area was defined according to the physician’s views of his or her practice as “urban”, “suburban” or “rural” (recoded as “urban” vs. “rural”). Some other variables were recoded in order to facilitate the analyses and the presentation of the results. The level of anxiety and the probability of seeing infected patients were measured by a scale from 0 (no anxiety) to 10 (major anxiety), and classified into low (≤2), moderate (3 to 6) and high (≥7). Variables with the following modalities: “Not at all”, “Not really”, “Yes, moderately” and “Yes, absolutely” were made as binary variables, with “Not at all” for “No” and the three other options for “Yes”. The level of information about the pandemic was evaluated through a score based on the perceived knowledge about four topics: the epidemic situation in China, the risk for the local population, the case definition and the management of suspected cases. Physicians were considered as poorly (n<2 topics), moderately (n=2-3) or highly (n=4) informed. Electronic surveys were built on LymeSurvey. An email with a link to the survey was sent to the physicians of the three sentinel networks, and a reminder was sent after one week. Electronic surveys were available from 14 to 27 February 2020 in France, from 19 to 28 February 2020 in Belgium and from 20 February to 2 March 2020 in Spain.

### Statistical analyses

Pearson’s chi-squared test and Fisher’s exact test were used for estimating the p-value of qualitative variables, and Kruskal-Wallis rank sum test for quantitative ones. The factors associated with the feeling of preparedness were studied using univariate and multivariate logistic regressions. The multivariate analysis was performed using a backward elimination procedure until all variables reached statistical significance (p≤0.05). Statistical analyses were performed using the R software version 3.5.0 [15].

### Ethics approval

This study was conducted in agreement with country-specific regulations on privacy and data collection and treatment. In addition, approvals by Ethical Review Boards or Committees were obtained when needed according to the country-specific regulations.

## Results

### Perception and preparedness of primary care physicians at the beginning of the COVID-19 pandemic

The electronic survey was filled by 35.2% (511/1450) of the investigated healthcare practitioners. Participating physicians were distributed as followed: 12.1% (n=62) from Belgium, 67.1% (n=343) from France and 20.8% (n=106) from Spain. Men represented 57.4% (n=292), median age was 56 years (IQR [42; 62]), and 70.7% (n=359) were working in urban areas (Table 1).

**Table 1:** Characteristics of primary care physicians by country

	Total N (%) N=511	Belgium N (%) N=62	France N (%) N=343	Spain N (%) N=106	p-value
**Participation**					
Targeted physicians	1450	98	1224	128	
Respondents	511 (35.2%)	62 (63.3%)	343 (28.0%)	106 (82.8%)	
**Type of physicians** (m.d.=0)					
General Practitioners	491 (96.1%)	62 (100%)	343 (100%)	86 (81.1%)	
Pediatricians	20 (3.9%)	0	0	20 (18.9%)	
**Sex** (m.d.=2)					
Female	217 (42.6%)	26 (41.9%)	131 (38.2%)	60 (57.7%)	<10^−2^
Male	292 (57.4%)	36 (58.1%)	212 (61.8%)	44 (42.3%)	
**Age (years)** (m.d.=9)					
25-39	106 (21.1%)	3 (4.8%)	100 (29.9%)	3 (2.9%)	<10^−5^
40-54	114 (22.7%)	9 (14.5%)	84 (25.1%)	21 (20%)	
≥ 55	282 (56.2%)	50 (80.6%)	151 (45.1%)	81 (77.1%)	
Median (IQR)	56 (42; 62)	61 (56; 65)	52 (37; 60)	60 (55; 62)	<10^−5^
**Practice area** (m.d.=3)					
Rural	149 (29.3%)	22 (35.5%)	79 (23%)	48 (46.6%)	<10^−4^
Urban	359 (70.7%)	40 (64.5%)	264 (77%)	55 (53.4%)	
**Physicians’ anxiety** (m.d.=7)					
Low anxiety feeling	176 (34.9%)	24 (38.7%)	140 (41.3%)	12 (11.7%)	<10^−5^
Moderate anxiety feeling	246 (48.8%)	28 (45.2%)	164 (48.4%)	54 (52.4%)	
High anxiety feeling	82 (16.3%)	10 (16.1%)	35 (10.3%)	37 (35.9%)	
Median (IQR)	4 (2; 6)	3 (2; 6)	3 (2; 5)	6 (4; 7)	<10^−5^
**Patients’ anxiety** (m.d.=4)					
Low anxiety feeling	107 (21.1%)	13 (21%)	73 (21.3%)	21 (20.4%)	0.85
Moderate anxiety feeling	286 (56.4%)	38 (61.3%)	188 (55%)	60 (58.3%)	
High anxiety feeling	114 (22.5%)	11 (17.7%)	81 (23.7%)	22 (21.4%)	
Median (IQR)	4 (3; 6)	5 (3; 6)	5 (3; 6)	4 (3; 6)	0.89
**Risk of seeing infected patients in the next 2 weeks** (m.d.=3)					
Low risk	400 (78.7%)	46 (74.2%)	295 (86%)	59 (57.3%)	<10^−5^
Moderate risk	97 (19.1%)	15 (24.2%)	46 (13.4%)	36 (35%)	
High risk	11 (2.2%)	1 (1.6%)	2 (0.6%)	8 (7.8%)	
Median (IQR)	1 (0; 2)	1 (1; 3)	1 (0; 2)	2 (1; 5)	<10^−5^
**Finding the measures taken by the health authorities suitable to limit the spread of COVID-19** (m.d.=3)	409 (80.5%)	51 (82.3%)	275 (80.2%)	83 (80.6%)	0.93
**Changes in professional practices** (m.d.=4)	207 (40.8%)	30 (48.4%)	124 (36.3%)	53 (51.5%)	<10^−2^
**Impact on consultations** (m.d.=3)	72 (14.2%)	8 (12.9%)	39 (11.4%)	25 (24.3%)	<10^−2^
** Types of consequences** (m.d.=5)					
Questions about any links with China during consultations	44 (61.1%)	4 (50%)	19 (48.7%)	21 (84%)	
Increased consultation time due to question about COVID-19	22 (30.6%)	1 (12.5%)	14 (35.9%)	7 (28%)	
Specific consultations for information on COVID-19	6 (8.3%)	0	1 (2.6%)	5 (20%)	
Phone calls on COVID-19	6 (8.3%)	2 (25%)	3 (7.7%)	1 (4%)	
Consultations of patients who thought they had contracted COVID-19	6 (8.3%)	1 (12.5%)	4 (10.3%)	1 (4%)	
**Anticipation of the epidemic arrival** (m.d.=3)	337 (66.3%)	40 (64.5%)	200 (58.3%)	97 (94.2%)	<10^−5^
** Types of anticipation measures** (m.d. =32)					
Search of guidelines	205 (60.8%)	33 (82.5%)	113 (56.5%)	59 (60.8%)	
Purchase of protection equipment	122 (36.2%)	4 (10%)	63 (31.5%)	55 (56.7%)	
Re-use of the influenza pandemic kits	106 (35.7%)	N.A.	80 (40%)	26 (26.8%)	
Office reorganization to avoid patients’ influx	60 (17.8%)	9 (22.5%)	34 (17%)	17 (17.5%)	
Other measures	13 (3.9%)	1 (2.5%)	9 (4.5%)	3 (3.1%)	
**Level of information regarding the epidemic** (m.d.=9)					
Low information level	80 (16.3%)	6 (9.6%)	59 (17.5%)	15 (14.6%)	0.05
Moderate information level	168 (33.5%)	14 (22.6%)	120 (35.6%)	34 (33,0%)	
High information level	254 (50.6%)	42 (67.7%)	158 (46.9%)	54 (52.4%)	
**Feeling prepared to face the epidemic** (m.d.=4)	229 (45.2%)	28 (45.2%)	139 (40.6%)	62 (60.2%)	<10^−2^

m.d.: missing data; IQR: interquartile range; N.A.: not available

No differences in sex, age or location of sentinel professionals were observed for Belgium and Spain between respondents and non-respondents. In France, the GPs who participated in the study were slightly younger compared to the French GPs (mean age of 50 vs. 53 years old respectively, p-value <10^−3^) and less urban (77% vs. 86% respectively, p-value <10^−3^). However, there was no difference in sex ratio (62% of men in the study vs. 59% among the French GPs, p-value=0.37).

**Fig. 1:**
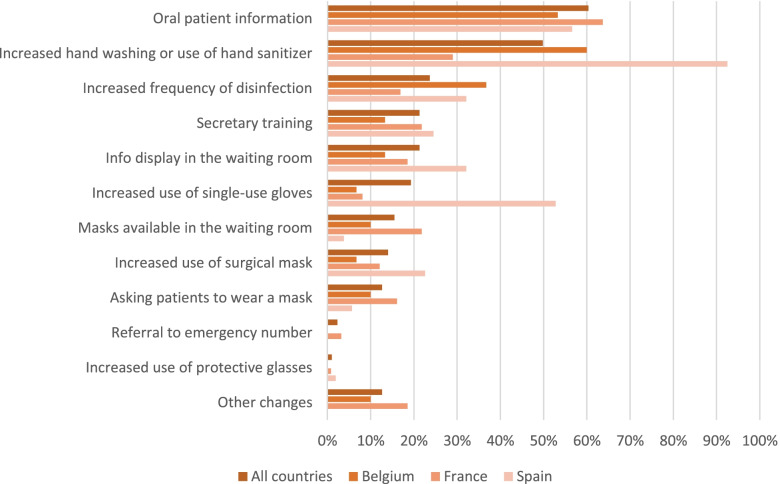
Professional changes by country at the beginning of the COVID-19 pandemic

Only 16.3% of physicians (n=82) and 22.5% (n=114) of patients were highly worried about the COVID-19 pandemic. The physicians were 80.5% (n=409) to find the measures taken by the health authorities appropriate to control its spread, and 40.8% (n=207) had made changes in their professional practices (Table 1). The most frequent changes were “increased oral information given to patients about COVID-19” (60.4%, n=125), “increased handwashing or hand sanitizing” (49.8%, n=103), and “increased frequency of disinfection” (23.7%, n=49) (Fig. 1). Physicians reported impacts on their consultations (like an increase in time) for 14.2% of them (n=72), and 66.3% (n=337) had started anticipating the epidemic arrival. The main anticipation measures were the research of action guidelines (60.8%, n=205) and the purchase of protection equipment (36.2%, n=122).

**Fig. 2:**
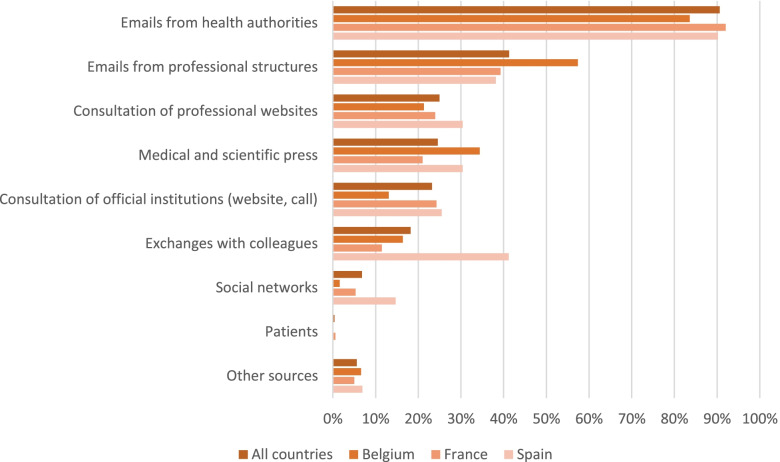
Source of information by country when facing a suspected case of COVID-19

Half of the physicians (50.6%, n=254) felt they received clear information from health authorities overall. For 90.6% (n=454), the main source of information consisted of emails sent by health authorities (Fig. 2).

### Factors associated with preparedness

Less than half of primary care physicians (45.2%, n=229) felt prepared for the epidemic arrival, ranging from 40.6% (139/342) in France to 60.2% (62/103) in Spain. Factors positively associated with feeling prepared were: being a Spanish practitioner (adjusted OR=4.34; 95%CI [2.47; 7.80]), being a man (aOR=2.57, 95%CI [1.69; 3.96]), finding the measures taken by authorities appropriate (aOR=1.72, 95%CI [1.01; 3.00]) and being highly informed (aOR=4.82, 95%CI [2.62; 9.19]). Factors negatively associated were: being moderately (aOR=0.34; 95%CI [0.21; 0.53]) or highly worried (aOR=0.27; 95%CI [0.14; 0.52]) (Table 2).

**Table 2:** Factors associated with the feeling of preparedness among primary care physicians (univariate and multivariate analyses)

		n	Feeling prepared	OR [95% CI] Univariate analysis	p-value	OR [95% CI] Multivariate analysis	p-value
Age	25-39	105	43 (41.0%)	Ref.	0.01		
	40-54	114	39 (34.2%)	0.76 [0.44;1.32]			
	≥ 55	278	142 (51.1%)	1.53 [0.97;2.42]			
Country	France	342	139 (40.6%)	Ref.	0.01	Ref.	<10^−5^
	Belgium	62	28 (45.2%)	1.2 [0.69;2.07]		0.94 [0.5;1.75]	
	Spain	103	62 (60.2%)	**2.21 [1.41;3.48]**		**4.34 [2.47;7.8]**	
Sex	Female	216	78 (36.1%)	Ref.	0.001	Ref.	<10^−4^
	Male	291	151 (51.9%)	**1.91 [1.33;2.74]**		**2.57 [1.69;3.96]**	
Type of practice area	Rural	149	69 (46.3%)	Ref.	0.74		
	Urban	358	160 (44.7%)	0.94 [0.64;1.38]			
Physicians’ anxiety	Low	176	104 (59.1%)	Ref.	<10^−4^	Ref.	<10^−5^
	Moderate	245	93 (38.0%)	**0.42 [0.28;0.63]**		**0.34 [0.21;0.53]**	
	High	82	31 (37.8%)	**0.42 [0.24;0.72]**		**0.27 [0.14;0.52]**	
Patients’ anxiety	Low	107	54 (50.5%)	Ref.	0.46		
	Moderate	285	126 (44.2%)	0.78 [0.5;1.21]			
	High	114	49 (43.0%)	0.74 [0.43;1.26]			
Risk of seeing infected patients in the next 2 weeks	Low	399	186 (46.6%)	Ref.	0.03		
	Moderate	97	35 (36.1%)	0.65 [0.41;1.02]			
	High	11	8 (72.7%)	3.05 [0.87;14.1]			
Practice changes	No	300	131 (43.7%)	Ref.	0.39		
	Yes	206	98 (47.6%)	1.17 [0.82;1.67]			
Impact on consultations	No	435	191 (43.9%)	Ref.	0.16		
	Yes	72	38 (52.8%)	1.43 [0.87;2.36]			
Feeling response measures appropriate	No	99	32 (32.3%)	Ref.	0.01	Ref.	0.05
	Yes	408	197 (48.3%)	**1.95 [1.24;3.14]**		**1.72 [1.01;3]**	
Level of information	Low	80	21 (26.2%)	Ref.	< 10^−5^	Ref.	< 10^−5^
	Moderate	167	46 (27.5%)	1.07 [0.59;1.98]		1.02 [0.54;2]	
	High	254	161 (63.4%)	**4.86 [2.82;8.67]**		**4.82 [2.62;9.19]**	
Anticipation	No	171	84 (49.1%)	Ref.	0.20		
	Yes	336	145 (43.2%)	0.79 [0.54;1.14]			

## Discussion

This study enabled to identify the initial risk perceptions and the feeling of preparedness among primary care physicians from Belgium, France, and Spain when COVID-19 pandemic emerged in Europe and when only isolated cases were observed in those three countries.

At the beginning of the COVID-19 pandemic, between mid-February and the beginning of March 2020, the majority of primary care physicians investigated were little to moderately worried about the disease. As suggested by the results of the study, this could be explained by a low perceived risk of handling infected patients, the feeling to be well-informed and the trust in health authorities’ ability to implement appropriate measures to limit the spread of the disease. Moreover, as the pandemic was still emerging in Europe, it appeared that the general population was not yet really concerned about its evolution and severity. About 20% of the patients followed by the physicians surveyed in the three countries were very worried about the COVID-19 pandemic, whereas this rate was around 30% among the general population over the pandemic course [16]. The global feeling of low to moderate anxiety, both for health professionals and the general population, could appear to be contradictory with the predominant feeling of the physicians of being insufficiently prepared to face the COVID-19 pandemic, as they were not directly involved in the management of this growing epidemic. Regarding the dramatic evolution of the pandemic in Europe in the weeks following the study, it appears that the scientific data available at this time and transmitted to the physicians could have given a wrong assessment of the spread and the severity of the disease [17, 18]. A previous study had highlighted that making available an internal information channel to ensure factual, accurate, and reliable information while preventing information overload represents a key measure in increasing infectious disease preparedness [19]. With the current hindsight on the pandemic, it seems essential to better integrate the primary care physicians into the information, training and protection channels for this kind of health risk, which may be lacking at this level, unlike the hospital level, and this is could be managed by the health authorities [20].

Differences between countries concerning the level of anxiety and the feeling of preparedness were observed, with Spanish physicians feeling more anxious (even more anxious than their patients) but more prepared, while Belgian and French physicians were little worried and moderately prepared. Such variations have been previously studied across countries, with Asian countries feeling more prepared than European or Northern American countries to face emerging diseases [13, 21]. However, no comparison between European countries has been undertaken. The national epidemic context was rather similar over the study period in the three countries involved in the study: 6 COVID-19 cases and 2 related-deaths were reported in France; 134 cases and no deaths in Spain; no cases or deaths in Belgium [2]. The main difference is that the study started in Spain later than in France, and was ended up one week later than in Belgium and France. The anxiety of the Spanish GPs could have been higher, as the European situation was complicated at that time (i.e. Italian situation). This contradictory feeling of anxiety and preparedness among Spanish physicians could be explained also by differences in organization and structure of the primary care services. In Spain, the primary care physicians are included in a complex structure of the public health system (including health administrative services, hospitals and primary care) that were contacted and informed periodically by the health authorities. While these contacts may have helped Spanish primary care professionals feel prepared, they may also have made the physicians anxious about the pandemic. Thus, communication regarding the epidemiological context could have been emphasized in Spain compared to Belgium and France, as well as the preparation of the health services, which could contribute to the higher feeling of preparedness of the Spanish GPs. A comparison could be of interest in adapting one country’s most effective public health measures in terms of information and communication to the other countries [21–23]

This study has some limitations. Physicians included are part of sentinel surveillance networks, which generate inherent biases (these professionals are more interested in research, well-informed and specially concerned with advances in clinical practice), making the results not representative of the primary care physicians in these countries. In France, the *réseau Sentinelles* representativeness has been previously studied, showing no particular differences regarding age and professional activities [10]. In Belgium, the Sentinel GPs have been selected to cover the whole country and form a representative sample of GPs in the country regarding age, sex and geographical distribution [11]. In Spain, *Red Centinela Sanitaria* evaluates representativeness yearly using cluster analysis and principal components analysis to ensure a good representation [12]. Even if the representativeness of the physicians participating in these three sentinel networks tries to be reached as much as possible, selection biases inherent to the present study could have occurred, linked in particular to the data collection process. Indeed, inclusion was made on a voluntary basis. However, this had no impact on the representativeness of the Belgian and Spanish physicians in terms of age, sex and location of practice. Regarding the differences observed in France in terms of age and location of practice, this does not seem to have influenced physicians’ feeling of preparedness or anxiety. Indeed, the perceptions of French participating GPs were similar to the ones of Belgian GPs. Finally, this cross-sectional study lacks a follow-up during this COVID-19 pandemic, which could have been particularly interesting to evaluate and adapt the guidelines and information campaigns. Repeating this study through the course of the COVID-19 pandemic, under different conditions of the epidemic and of awareness, could help identify critical aspects to be further improved.

## Conclusions

The present study could help health authorities to define preparedness planning for primary care physicians against an emerging epidemic, and identify areas of improvement in terms of information and actions.

## Supplementary Information

**Additional file 1.** Physicians’ questionnaire on early perception of the COVID-19 pandemic.

## Data Availability

Data will be available from the corresponding author on reasonable request. Requests will be submitted to the scientific committees of each country. Data provided will have to respect the constraints on privacy and treatment imposed by the national regulatory authorities.

## Abbreviations

GPs: General practitioners
aOR: Adjusted odds ratio
CI: Confidence interval
