# Supplementary material for: Initial risk perception and feeling of preparedness of primary care physicians regarding the COVID-19 pandemic in Belgium, France and Spain in February 2020
Source: BMC Prim Care. 2022 Jan 23;23:18. doi: 10.1186/s12875-021-01588-5 (PMC8784169; doi:10.1186/s12875-021-01588-5)
Supplement: Supplementary file 1 — Additional file 1. Physicians’ questionnaire on early perception of the COVID-19 pandemic. [file 12875_2021_1588_MOESM1_ESM.pdf]

Additional file 1. Physicians' questionnaire on early perception of the COVID-19 pandemic

1. What is your specialty?

- General Practitioner **1**
- Paediatrician **2**

2. What is your age? *2-digit whole number*

3. What is your gender?

- Female **1**
- Male **2**

4. In which region do you practice? *Select from picklist of regions. Regions were adapted to the countries.*

5. Which of the following best describes your practice area?

- Urban **1**
- Suburban **2**
- Rural **3**

6. Are you worried about the Covid-19 coronavirus (ex 2019-nCoV) epidemic?
To answer this question, please use a note between 0 and 10 (note 0 indicating that you are not worried at all and note 10 that you are very worried)

7. Does the Covid-19 coronavirus (ex 2019-nCoV) epidemic worry your patients?
To answer this question, please use a note between 0 and 10 (note 0 indicating that they are not worried at all and note 10 that they are very worried).

8. In your opinion, what is the probability of seeing a patient infected with the Covid-19 coronavirus (ex 2019-nCoV) in consultation in the next two weeks?
To answer this question, please use a note between 0 and 10 (note 0 indicating that this risk is very low and note 10 that it is very high).

9. Since the start of the epidemic, have you changed your professional practices due to the emergence of the Covid-19 coronavirus (ex 2019-nCoV)?

- Yes, absolutely **4**
- Yes, moderately **3**
- No, not really **2**
- No, not at all **1**
- I don't know **99**

9b. *(if "Yes, absolutely", "Yes, moderately" or "No, not really" to question 9)* **What modification(s) in your professional practices have you made?** *(Select all answers that apply)*

- Increased frequency of disinfection of your practice and / or equipment **1**
- Increased your frequency of wearing an anti-splash surgical mask **2**
- Increased your frequency of wearing protective glasses **3**
- Provision of anti-splash surgical masks in the waiting room **4**

- Increased frequency of asking your patients to wear an anti-splash surgical mask in the waiting room or during the consultation **5**
- Increased frequency of hand washing or use of alcohol-based solute **6**
- Increased frequency of wearing single-use gloves **7**
- Information display in the waiting room **8**
- Oral patient information during consultations **9**
- Training of secretaries on the Covid-19 coronavirus **10**
- *Increased referral of patient (s) to the 15* ***11*** *(France specific item. It is the phone number to the service meant to call to report a suspect COVID-10 cases.)*
- Increased referral of patient (s) to the emergency room **12**
- Other (please specify)*(free text)* **13**

10. Since the start of the epidemic, has the Covid-19 coronavirus (ex 2019-nCoV) had any repercussions on the organization of your consultations?

- Yes, absolutely **4**
- Yes, moderately **3**
- No, not really **2**
- No, not at all **1**
- I don't know **99**

10b. *(if "Yes, absolutely", "Yes, moderately" or "No, not really" to question 10)* **In what consisted these repercussions?** *(Select all answers that apply)*

- Patients consulting specifically to obtain information on the Covid-19 **1**
- Patients consulting because they think they might have contracted Covid-19 **2**
- Increased consultation time due to questions asked by patients on Covid-19 **3**
- Receiving phone calls to inquire about Covid-19 **4**
- Asking questions about any links with China (return from a trip or link with a traveller returning from China in the last 2 weeks) in your interview of patients consulting for an ARI **5**
- Other (please specify) *(free text)* **6**

11. Do you consider that you have received sufficiently clear information from the health authorities regarding the coronavirus epidemic in China?

- Yes, absolutely **4**
- Yes, moderately **3**
- No, not really **2**
- No, not at all **1**
- I don't know **99**

12. Do you consider that you have received sufficiently clear information from the health authorities regarding the risk associated with this coronavirus for the *Belgian/French/Spanish* population today?

- Yes, absolutely **4**
- Yes, moderately **3**
- No, not really **2**
- No, not at all **1**
- I don't know **99**

13. Do you consider that the definition established by the health authorities of a suspected case of infection with Covid-19 coronavirus (ex 2019-nCoV) is sufficiently clear?

- Yes, absolutely **4**
- Yes, moderately **3**
- No, not really **2**
- No, not at all **1**
- I don't know **99**

14. Do you consider that you are sufficiently informed by the health authorities about the action to be taken if confronted with a suspicious case for Covid-19 coronavirus (ex 2019-nCoV)?

- Yes, absolutely **4**
- Yes, moderately **3**
- No, not really **2**
- No, not at all **1**
- I don't know **99**

15. How were you informed of these action guidelines? *(Select all answers that apply)*

- Emails (distribution lists, newsletters, etc.) sent by health authorities **1**
- Spontaneous consultation on your part of official institutions (website, telephone call) **2**
- Emails received from your professional structures (unions, learned societies, etc.) **3**
- Spontaneous consultation on your part of professional websites **4**
- Exchanges with other colleagues **5**
- Medical and scientific press **6**
- Social networks **7**
- By the patients **8**
- Other (please specify)(*free text*) **9**
- I am not informed at all *(exclusive response, if selected, no other response is possible)* **10**

16. Do you consider that the measures taken during the last two weeks by the health authorities to limit the spread of the epidemic in *Belgium/France/Spain* are appropriate?

- Yes, absolutely **4**
- Yes, moderately **3**
- No, not really **2**
- No, not at all **1**

17. Do you feel prepared, if the coronavirus Covid-19 (ex 2019-nCoV) epidemic were to occur in *Belgium/France/Spain*?

- Yes, absolutely **4**
- Yes, moderately **3**
- No, not really **2**
- No, not at all **1**
- I don't know **99**

18. Are you starting to anticipate a possible coronavirus Covid-19 (ex 2019-nCoV) epidemic arrival in *Belgium/France/Spain*?

- Yes, absolutely **4**
- Yes, moderately **3**
- No, not really **2**
- No, not at all **1**
- I don't know **99**

18b. (if “Yes, absolutely”, “Yes, moderately” or “No, not really” to question 18) **How do you begin to anticipate it?** (select all answers that apply)

- Purchase of protection equipment **1**
- Research of guidance on the actions to be taken **2**
- Reorganisation of the practice to avoid the influx of contagious patients **3**
- Reuse of the influenza pandemic kits sent in 2009 **4** *(If applicable in the country)*
- Other (please specify)*(free text)* **5**

19. Below you can suggest tools that could improve the information you receive, facilitate the potential management of cases in ambulatory care, or even follow-up on the disease.
Free text

20. Do you think that the importance given and the place taken in the media and in the society of this epidemic is excessive?

- Yes, absolutely **4**
- Yes, moderately **3**
- No, not really **2**
- No, not at all **1**
- I don't know **99**

21. Is there something you would like to add?
Free text
